# Multitarget Phytocomplex: Focus on Antibacterial Profiles of Grape Pomace and *Sambucus ebulus* L. Lyophilisates Against Extensively Drug-Resistant (XDR) Bacteria and In Vitro Antioxidative Power

**DOI:** 10.3390/antibiotics13100980

**Published:** 2024-10-17

**Authors:** Vladimir S. Kurćubić, Vesna Đurović, Slaviša B. Stajić, Marko Dmitrić, Saša Živković, Luka V. Kurćubić, Pavle Z. Mašković, Jelena Mašković, Milan Mitić, Vladimir Živković, Vladimir Jakovljević

**Affiliations:** 1 Department of Food Technology, Faculty of Agronomy, University of Kragujevac, Cara Dušana 34, 32102 Čačak, Serbia; 2 Department of Biology, Microbiological Biotechnology and Plant Protection, Faculty of Agronomy, University of Kragujevac, Cara Dušana 34, 32102 Čačak, Serbia; djurovicvesna@yahoo.com; 3 Faculty of Agriculture, University of Belgrade, Nemanjina 6, 11080 Belgrade, Serbia; stajic@agrif.bg.ac.rs; 4 Veterinary Specialized Institute “Kraljevo”, Žička 34, 36000 Kraljevo, Serbia; markodmitric@gmail.com (M.D.); sasha98@live.se (S.Ž.); 5 Department of Medical Microbiology, University Clinical Center of Serbia, Pasterova 2, 11000 Beograd, Serbia; kurcubiclk@gmail.com; 6 Department of Chemical Engineering, Faculty of Agronomy, University of Kragujevac, Cara Dušana 34, 32102 Čačak, Serbia; pavlem@kg.ac.rs (P.Z.M.); jelenav@kg.ac.rs (J.M.); 7 Faculty of Science and Mathematics in Niš, University of Niš, Višegradska 33, 18000 Niš, Serbia; milanmitic83@yahoo.com; 8 Department of Physiology, Faculty of Medical Sciences, University of Kragujevac, 69 Svetozara Markovica St., 34000 Kragujevac, Serbia; vladimirziv@gmail.com (V.Ž.); drvladakgbg@yahoo.com (V.J.); 9 Department of Human Pathology, Sechenov First Moscow State Medical University, 8 Trubetskaya St., 119991 Moscow, Russia

**Keywords:** by-product, weeds, plant extracts, bioactive molecules, antibacterial activity, antioxidant activity, synergistic effect

## Abstract

**Objectives:** This study was conceived with the aim of translating the experience and knowledge of the research group into the design and creation of multi-active phytocomplex cocktails from lyophilised winery by-products (Grape Pomace—GP) and weeds (*Sambucus ebulus* L., Dwarf Elder—DE). **Methods:** Quantification of bioactive molecules was performed by high-performance liquid chromatography (HPLC) method. **Results:** In the extract obtained from lyophilised GP, the most dominant component that was quantified was petunidin-3-glucoside. Prominent compounds that were quantified in DE extract were cyanidin derivatives. The total number of microorganisms in lyophilisates is low, but some of them still survive lyophilisation. Antibacterial activity was determined by microdilution, the minimum inhibitory concentration (MIC) of the tested bacteria ranged from 0.78 mg/mL to 25.00 mg/mL. Antibacterial susceptibility testing (AST) revealed that *Klebsiella* spp. and *Acinetobacter baumannii* complex are extensively drug-resistant (XDR). **Conclusions:** The GP + DE cocktail showed very strong AB power against both tested XDR bacteria. The total phenolic content and antioxidative effect (determined spectrophotometrically) indicate their linear correlation.

## 1. Introduction

In today’s fast-paced life, preventive lifestyle changes (or those in support of pharmacotherapy) are necessary to maintain health. A more “moderate” lifestyle and striving for a healthier way of life can delay the application of pharmacotherapy, and can be upgraded and completed by taking nutraceuticals [1].

Nutraceuticals are defined as a food or part of a food that has a positive effect on human well-being, and whose health benefits have been tested and proven. Today, patients and consumers in general pay a lot of attention to phytotherapeutics (or herbal medicines), which are becoming increasingly popular [2].

Plants have GRAS status (GRAS—Generally Recognised as Safe) and 1340 of them have been registered as containing bioactive molecules, with a plethora (over 30,000) of antimicrobial compounds extracted from them [3,4].

Reformulation strategies strengthen and ensure the willingness of consumers to pay a higher price for their own requirements for the naturalness of food without synthetic additives, marked as “clean label”. Accordingly, when it comes to food production (the largest part of the market for natural plant extracts), subjects in the food business have begun to evaluate the use of environmentally friendly and consumer-friendly additives of natural origin, instead of synthetic ones [5,6].

Phytotherapy is constantly being intensively improved, and, more recently, great hopes have been placed on the combined effect of a mixture of ingredients of one or a cocktail of several phytocomplexes. A multidisciplinary and multifunctional approach and direction in the research and development of phytocomplexes for the needs of cosmetics, pharmaceuticals and the food industry, as well as a multitarget therapeutic approach in medicine and nutrition, offer new possibilities for the prevention and treatment of various conditions, disorders and diseases, primarily those of known etiology, defined prepathological condition and complex genesis [1,2]. Secondary plant metabolites play an extremely important role in pharmaceuticals—more than 30% of conventional drugs originate directly or indirectly from natural products [7].

Conventional therapeutics solves one problem at a time, while phytotherapy hits multiple targets. In order for such hits to make sense, it is imperative to discover and learn about the mechanisms of potentiation, i.e., the synergistic effect between the bioactive molecules in them, in order to achieve the goal of phytotherapeutics exhibiting greater effects than the sum of the effects of individual components. Contemporary thinking and research indicate the importance of the position that fractionation based on bioactivity can result in a significant reduction or loss in activity. Extraction is the most important step when analysing bioactive compounds from plant materials. Compared to conventional extraction methodologies, where often the quality, quantity and time of extraction are questionable, green extraction methods increase the extraction efficiency and reduce the extraction time [4,8,9,10,11,12].

Despite the intensive development of green extraction methods and attempts to achieve fast, economical and complete isolation of bioactive molecules from plant material, and to make the whole process and product eco-friendly and consumer-friendly at the same time, other researchers propose the use of plant raw materials without prior extraction process. Crude extracts of selected plants are prepared with polar and non-polar solvents, so regardless of the variable extraction profile for each plant during the test, the total polar solvents performed the extraction as efficiently as possible [13].

Lyophilisation, i.e., freeze drying, is widely used in the production of pharmaceuticals because it improves their stability and enables long-term storage of drugs that are usually labile due to their composition. During lyophilisation, no chemical substances are used, and the highest level of bioactive ingredients is delivered to consumers through safe, environmentally friendly and different products [14]. Freeze drying is the superior non-convective drying of different materials by sublimation, by direct transition from solid to gaseous state. The frozen product is dried under a vacuum, without thawing, which preserves the plant’s raw materials without degrading the bioactive compounds in them, with minimal changes in color, odor and taste [15,16]. There are a number of studies that confirm the superiority of lyophilisation in terms of properly protecting the quality and quantity of bioactive molecules and volatile substances originating from raw plant materials or in other matrices [14,15,16,17,18,19,20,21,22,23,24,25,26,27,28,29,30,31,32,33,34], with few studies that observed certain negative effects of lyophilisation [18]. Existing research points to certain disadvantages of the process of preserving different substrates by lyophilisation: it reduces the effect of certain medicinal plants, it cannot completely preserve volatile substances, phenols and carotenoids, and it increases the rate of mutations in unicellular organisms; therefore, pharmacological and clinical studies must be carefully prepared and performed [18]. The modern view is that lyophilisation represents the “gold standard” for drying plant extracts because it protects their quality in a superior manner and extends their shelf life [35]. The advantages of the lyophilisation method outweigh its disadvantages.

An example of the use of weed plants can be found in the scarce but interesting descriptions in the literature about the characterisation/use of the weed plant Dwarf Elder (DE-*Sambucus ebulus* L. fam. Caprifoliaceae), present all over the world, including Serbia. Traditional and folk medicine uses it for multiple purposes, e.g., externally/internally against rheumatism [36,37,38]. The fruit contains valerian, malic and tartaric acids, tannins, anthocyanins (the blue color of the juice) and pectins. The whole plant acts as a purgative and a diuretic and causes sweating. Immunostimulatory, anti-inflammatory, antioxidant and anti-cancer effects come from polyphenols, flavonoids and anthocyanins [39], while berries are a valuable source of nutrients [40]. Other authors described in their comprehensive review a number of roles of *Sambucus ebulus* preclinical studies in biomedical research: antioxidative, anti-inflammatory, analgesic, antimicrobial, anticancer, wound healing, antidepressant, antigiardial, scolicidal and neuroprotective effects. Descriptions of clinical studies are linked to paederus dermatitis, metabolic disorders and knee osteoarthritis [41].

In the case of Grape Pomace (GP), which is included in our study, this approach allows for more complete reuse of winery leftovers and enables intensive enrichment of certain products with fibers, minerals, proteins, oils and other GP constituents, such as phenols, including non-extractable phenolic compounds. In this way, the nutritional value and potential health benefits can be improved. If extraction is not applied, the process of obtaining specific powder products is more economical, with no negative consequences for the environment (use of organic solvents during extraction), which is a sustainable approach [42]. It is extremely important to eliminate the huge amounts of GP that are generated in wineries and thus solve several serious waste management problems that have emerged as a result of open area discharge of GP leftovers. Also, waste can be turned into products with added value through adequate procedures, thus encouraging a circular economy [9,43,44,45,46,47,48,49,50,51]. The food waste was reduced, facilitating a circular economy model, as well as simultaneously presenting environmental and economic advantages.

Getting to know and describing the interactions of individual bioactive molecules that enable synergistic effects in bioactive phytocomplexes is of invaluable importance because the mechanisms of interactions are often subject to change or remain unknown.

DE has 40% higher phenolic content than other *Sambucus* species [52]. Short-term heating can eliminate toxic compounds (cyanogenic glycosides and lectins), without loss or damage to total phenols. Berries contain pigments that researchers added to pastry spreads/creams, sweets, dough, jams, liqueurs and wine as a coloring agent [53].

In our study, we presented a multitude of different reviews or original scientific results and reports that include in vitro, in vivo and clinical evidence of the effectiveness of various procedures and approaches of designed and created phytocomplexes, which indicate a wide range of potential applications and impacts, as well as their potential cost-effectiveness. Researchers from all meridians point to the necessity of testing plant raw materials as natural sources of a plethora of phytochemicals, primarily for toxicity and microbiological safety [54,55,56,57,58,59].

By sublimating the experience and knowledge of the research team, along with the latest knowledge in the creation of natural ingredients, natural antimicrobial agents and antioxidants for various purposes, we formed the goal of our research: creating cocktails of lyophilised agri-food sector by-products and weeds (Grape Pomace—GP and *Sambucus ebulus* L., Dwarf Elder—DE), i.e., production of multi-active phytocomplexes [59,60,61,62,63,64,65]. The mixture of phytocomplexes was designed based on the knowledge of the total phenolic content, and antimicrobial and antioxidative activity for the two selected plant raw materials (GP and DE), based on the displayed effects, after which these activities were tested in their 50%:50% mixture, within the phytocomplex. In the GP/DE phytocomplex cocktail, additional activity was tested against proven XDR microorganisms, due to the exceptional importance of reduced use of synthetic antibiotics and the imminent emergence of antibiotic resistance. This study is interesting since there is no available data from the literature on the chemical composition and antibacterial and antioxidative activities of the mentioned phytococtail.

## 2. Results and Discussion

### 2.1. Phytochemicals Compounds in Lyophilisates Obtained from GP and DE

Table 1 gives the relevant analytical parameters necessary for the identification of selected phenolic compounds, including a list of relevant analytical standards.

**Figure 1:**
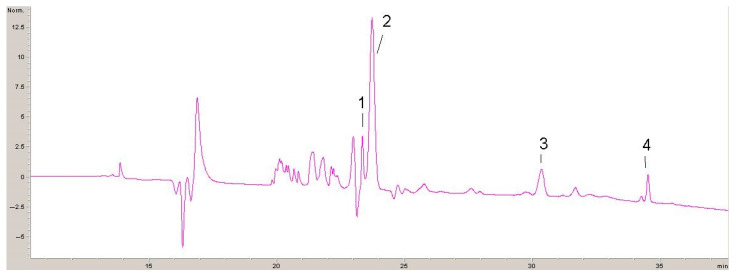
HPLC chromatogram recorded at 520 nm for GP: 1—petunidin-3-glucoside; 2—malvidin-3-glucoside; 3—malvidin-acetyl-glucoside; 4—malvidin-coumaroyl-glucoside.

**Figure 2:**
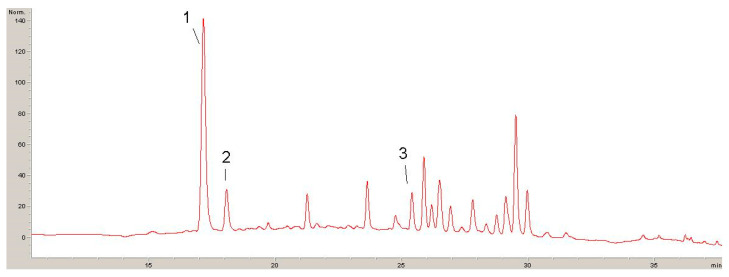
HPLC chromatogram recorded at 320 nm for DE: 1—caffeic acid; 2—chlorogenic acid; 3—*p*-coumaric acid.

**Figure 3:**
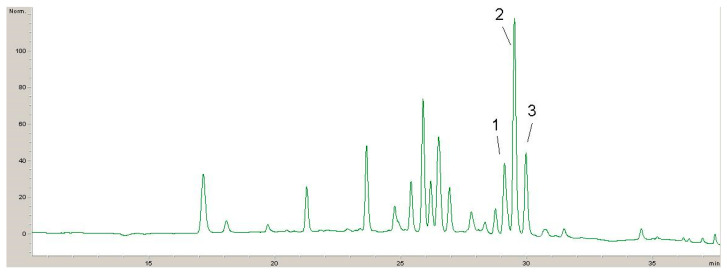
HPLC chromatogram recorded at 360 nm for DE: 1—rutin; 2—hyperoside; 3—isoquercetin.

**Figure 4:**
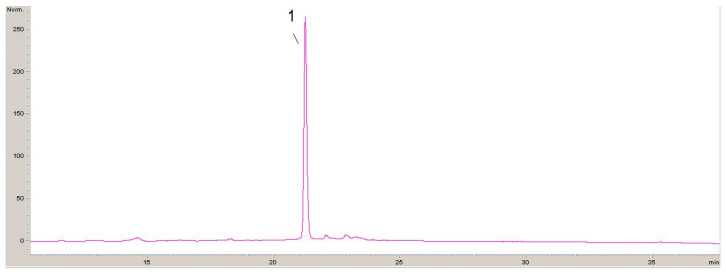
HPLC chromatogram recorded at 520 nm for DE 1-cyanidin-3-glucoside.

Four chromatograms recorded at 320 nm, 360 nm and 520 nm with identified phenolic compounds are shown below (Figure 1, Figure 2, Figure 3 and Figure 4).

Results of the polyphenolic composition of the by-product extract of the winery GP are given in Table 2.

The quantitative analysis of the composition of polyphenolic compounds of extracts of GP revealed the presence of malvidin-3-glucoside, malvidin-acetyl-glucoside, petunidin-3-glucoside and malvidin-coumaroyl-glucoside. The most dominant quantified component is malvidin-3-glucoside at a concentration of 2388 mg/g extract (74.9% of the total amount of polyphenolic compounds proven found in the Grape Pomace extract). Malvidin-acetyl-glucoside, petunidin-3-glucoside and malvidin-coumaroyl-glucoside were also quantified in lower but very significant concentrations (about 25% of the total amount of polyphenolic compounds detected in the Grape Pomace extract).

The phenolic composition of various food products fortified with GP was reported in the most recent study [43], whereby the tubular food matrix model showed grape variety, the vegetative structure of GP, GP processing, GP extract composition, total polyphenols content and non-extractable phenolic compounds.

A remarkable tabular presentation reveals 16 non-extractable phenolic compounds quantified in GP, which belong to the groups of phenolic acids, flavonoids and stilbene in the investigation of the influence of in vitro gastrointestinal digestion on composition, antioxidative activity and the bioaccessibility of GP phenolic extract. The highest content of flavonoids (catechin, rutin, quercetin, taxifolin, kaempferol, naringenin and apigenin) were recorded in the extract before digestion, whose concentrations ranged from 3.43 to 28.26 mg/g in dry GP [12]. An outstanding review was recently published that compared different approaches that should enable more focused research into better and safer resveratrol treatment options [66].

Extract of GP (white grape) had a higher content of total polyphenols, flavonoids and phenylpropane derivatives (37.80, 1.89 and 17.64 mg/g, respectively) than red grape extract (32.00, 0.54 and 9.11 mg/g, respectively). The lowest concentrations of these bioactive molecules were determined in grape cane extract (18.45, 0.33 and 1.86 mg/g, respectively) [67].

Phenolic biomolecules and radical-scavenging activity by the DPPH method in GP obtained in the production of wine from red wine grapes of the Vranac variety (*Vitis vinifera* L.) were determined and the possibility and validity of their potential application for different purposes were assessed [68]. By comparing the results of the group of authors [68] with the results of our research, we can state that the content of identified glycosides in our GP samples is slightly higher.

The results of the polyphenolic composition of the extract of plant species DE are presented in Table 3.

Quantitative analysis of the composition of non-extractable phenolic compounds in the extract of the plant species *Sambucus ebulus* L. revealed the presence of caffeic acid, chlorogenic acid, rutin, hyperoside (que-galactoside), isoquercetin (que-glucoside) and cyanidin derivative. The most dominant component that was quantified was cyanidin derivatives in a concentration of 3.730 mg/g of the extract, which occupies as much as 43.4% of the total amount of tested non-extractable phenolic compounds present in the extract.

According to the data from the literature, the predominant anthocyanin in DM extracts is cyanidin-3-galactoside, followed by cyanidin-3-sambubioside [69]. Other cyanidin glycosides reported include cyanidin-3-glucoside, cyanidin-3,5-diglucoside, cyanidin-3-rutinoside and cyanidin-3-arabinoside. Osman et al. [70] reported that using HPLC/MS led to the identification of five major anthocyanins. These were cyanidin 3-O-sambubioside-5-O-glucoside and cyanidin 3,5-O-diglucoside (4), cyanidin 3-O-sambubioside, cyanidin 3-O-glucoside and cyanidin 3-O-rutinoside (5), with cyanidin 3-O-sambubioside as the most abundant anthocyanin, representing more than half of the total anthocyanin content.

Hyperoside (que-galactoside) was also quantified in a concentration of 2.319 mg/g extract, which occupies about 27% of the total amount of tested polyphenolic compounds present in the extract of the plant species *Sambucus ebulus* L. All other quantified components (caffeic acid, chlorogenic acid, rutin and isoquercetin (que-glucoside) were quantified in smaller but very significant concentrations and make up only about 29.6% of the total amount of tested non-extractable phenolic compounds present in the extract of the plant species *Sambucus ebulus* L. Comparative analysis of our research with the research of Păvăloiu et al. [71], we can conclude that the same polyphenolic components were identified and quantified with almost the same concentrations. This indicates the dominance of active components.

Chromatographic tests of the extracts of GP and DE established the existence of polyphenolic compounds. These phenolic compounds belong to different classes, starting with phenolic acids, flavonoids as well as various other compounds that are derivatives of the aforementioned classes of compounds such as esters, glycosides, etc. Extracts of plant species GP and DE have a diverse composition. The applied extraction procedure has a great influence on the composition of the extract, and therefore also affects the biochemical activities [72]. We assume that the reason for this phenomenon lies in the existence of different mechanisms that take place during heat and mass transfer, but also in the different solubility of the components in the solvent we used. The absence of phenolic acids in the GP extract can be explained by the decomposition of the compound that occurs during the extraction process itself and exposure to the effects of increased heating because it has been noted that heating causes the decomposition of some phenolic acids and the creation of highly reactive hydroxyl radicals within the gas bubbles [72]. On the other hand, we believe that the absence of caffeic and chlorogenic acid in the GP extract, which is present in the DE extract, is due to their low solubility under the conditions under which the extraction is performed. It is well known that the polarity of water decreases with increasing temperature. Therefore, under the given experimental and research conditions, less polar compounds should be better extracted. Differences in the content of total phenolic content determined by the spectrophotometric method and the HPLC-DAD method may occur as a result of the Folin–Ciocalteu reagent, which has the property of reacting with many groups of compounds and the interference of other compounds, which is also confirmed by works in this area [73,74].

Quantitative analysis of the polyphenolic compound composition of phytocomplex GP + DE extracts revealed the presence of caffeic acid, chlorogenic acid, *p*-coumaric acid, rutin, hyperoside (que-galactoside), isoquercetin (que-glucoside), cyanidin-3 glucoside, petunidin-3 glucoside, malvidin-acetyl-glucoside and malvidin-coumaroyl-glucoside. The most dominant component that was quantified was cyanidin-3-glucoside, petunidin-3-glucoside and hyperoside (que-galactoside) in a concentration of 4.134 mg/g extract, 2.348 mg/g extract and 2.454 mg/g extract, which occupies as much as 73.5% of the total amount of investigated polyphenolic compounds present in the extract of phytocomplex GP + DE. The results obtained by HPLC for phytocomplex GP + DE are in very good synergy with the results of testing separate extracts of GP and DE. The presence of the same components was observed in all samples, but the highest content of these components was in phytocomplex in GP + DE. Table 4 gives the results of the polyphenolic composition of phytocomplex GP + DE.

### 2.2. Bacterial Contamination (Microbiological Load) in Samples of Various Plant Lyophilisates

Based on Table 5, it can be concluded that there was no significant microbiological contamination of the lyophilised samples. The highest number of aerobic colonies was found in DE (1700 CFU/g). The lyophilised DE sample was contaminated with 60 CFU/g of yeast, while other microorganisms tested were either not isolated or their numbers were below the detection limit of the method. Useful information is also provided by the measured mean value of water activity for samples GP and DE, presented in Table 6. Significantly (*p* < 0.05) lower a_w_ values were determined in DE samples compared to GP. However, a_w_ values in both samples were relatively low, providing good stability. The correlation of a_w_ values with the microbial load in samples of various plant lyophilisates indicates the following: a_w_ values are very low (extreme microbiological stability of the matrix) and the total number of microorganisms is low, but they still survive lyophilisation. We believe that it is justified and expedient to think about and establish control and critical control points in the production of ready-to-use lyophilised food (as long as there is no additional processing).

### 2.3. AB Effects

GP polyphenols have been shown to affect the integration of G+ bacterial cell walls and degenerate G− bacterial outer membranes [87]. Ghendov-Mosanu et al. [88] determined the bactericidal effect of GP extract on Gram-positive bacteria (G+), such as *Bacillus subtilis* (ATCC 6633) and *Staphylococcus aureus* (ATCC 25923) and AB activity on *Escherichia coli* (ATCC 25922), through the action of polyphenols that destabilise the cytoplasmic membrane by changing its permeability and inhibiting the synthesis of nucleic acids in G− and G+ bacteria. There are projects and research to create functional packaging with the incorporation of bioactive molecules from GP as possible shelf-life enhancers. Recent research has indicated the possible development of bactericidal isotactic polypropylene (PP) by applying GP extracts, to ensure that the packaging provides lower water vapor permeability and AB activity against pathogens such as *E. coli* and *B. subtilis* [89]. In our study, lyophilisate extracts of GP and DE showed high AB activity on *E. coli* and *B. subtilis* (6.250 mg/mL), thus showing a good potential for use as natural food preservatives.

Applications of bioactive molecules from GP in medicine are of great importance [90,91]. Microbiological testing reveals that bioactive compounds within the GP matrix largely prevent uncontrolled pathogen proliferation while simultaneously promoting commensal growth in cell culture and small animal studies. A diverse gut microbiota results in improved gut health and function. GP also protects probiotic strains of bacteria from adverse conditions in the upper gastrointestinal tract, allowing them to reach the colon, indicating the enormous potential of GP in a prebiotic/probiotic cocktail to improve gut health in humans. The significant results observed in animal models indicate the need for additional human studies to successfully evaluate additional applications of GP, such as the delivery of synergistic combinations with commercially available probiotics likely to grow on prebiotics naturally found in GP. Well-designed clinical studies using chemically defined and purified GP fractions will allow the differentiation of the contribution of individual bioactive molecules [92].

Synergism is an understudied mechanism by which bioactive molecules of plant origin exert their AB efficacy. Today, scientists conduct their research using three main approaches to exploit the powerful synergistic properties of plant-derived AB molecules: (1) synergistic interactions of plant bioactive molecules with antibiotics; (2) synergistic combinations of herbal bioactive compounds; and (3) synergy of herbal bioactive molecules with nanomaterials [93].

The use of bioactive molecules from medicinal plant extracts as appropriate antimicrobial agents is important and should be supported by new metabolomic technologies to select the most effective combinations for application. Understanding the nature of interactions between bioactive molecules originating from different medicinal plants would greatly facilitate the development of modern and insightful combinations of antimicrobial therapies. The chances that the results of such studies will be current in terms of patents and their application in medical use are amplified by the fact that the modern approach to this area of research states that complex mixtures of bioactive molecules (phytocomplexes) are more effective than purified bioactive molecules precisely because of the synergistic effects of combined interactions [94]. It is an important fact that often synergistic compounds do not have bioactivity by themselves, but enhance the activity of bioactive molecules when they are combined [95].

Confusion about the categorisation of antagonistic, additive or synergistic interactions arises mainly due to the use of different reference models that should define the “expected” outcome of a given mixture of bioactive molecules from plant material. The most frequently used reference models, as well as their biological hypotheses and limitations, are presented in two contemporary comprehensive overviews. Advances in big data approaches offer the identification and description of bioactive molecules from a mixture of phytocomplexes, the recognition of the nature of their interactions and the elucidation of mechanisms of action that are continuously being investigated [96,97].

Antibacterial (AB) activity was tested by the microdilution method (minimum inhibitory concentrations and minimum bactericidal concentrations—MIC/MBC determination), using G+ and G− bacteria. As a positive control, MHB with bacterial inoculums was used, and as a negative control, MHB (sterility control). The results of the aforementioned analyses are shown in Table 7.

The MIC of the tested samples ranged from 0.78 mg/mL to 25.00 mg/mL. The strongest AB activity was shown by the GP sample with MIC values of 0.78 mg/mL against the bacteria *E. faecalis*. *L. monocytogenes* and *S. aureus* showed very strong sensitivity to plant extract obtained from lyophilised GP, with an MIC value of 3.125 mg/mL. GP showed moderately strong activity against *B. subtilis* and *S. enteritidis* (MIC 6.250 mg/mL). GP has a moderate effect against bacteria in the *Proteus* genus (12.500 mg/mL). Almost identical sensitivity to DE was shown by all tested bacteria, the strongest by *S. aureus* and *E. faecalis*, with an MIC value of 3.125 mg/mL. On all other tested bacterial strains, DE showed activity identical to GP.

Abdallah et al. [93] gave an extremely useful overview of the mechanisms of antibacterial agents of plant origin and the main phytochemical classes with potent AB activity, illustrating it in a table that shows certain plants with AB potential (in vitro) against pathogens that have been reported by the WHO in the last four years on the priority list. In our research, we used almost all of these microorganisms to test the effect of our plant extracts and phytocomplex cocktails from GP and DE.

The AB effects of GP polyphenols were tested in combination with a probiotic to evaluate the growth of pathogenic microorganisms such as *E. coli*, *Bacillus megaterium* and *L. monocytogenes*. The probiotic *Lactiplantibacillus plantarum* (*L. plantatum*) showed an increase of one logarithmic cycle after 24 h of incubation with GP; moreover, pathogenic microorganisms were inhibited by the synergistic action of GP and *L. plantatum*, reduced by three logarithmic cycles [90].

Ethanol extract from DE leaves displays a significant inhibition of δ-hemolysin production in methicillin-resistant *S. aureus* (MRSA) MDR isolates, thus proving an impressive AB activity [36,98].

The tested *V. vinifera* extracts had a noticeable AB effect on several bacterial strains, normal inhabitants of the oral microbiota or members of the periodontopathic microbiota. An in vitro study confirmed the positive effects of including a combined extract (a mixture of equal parts of single extracts) in the formulation of lyophilised mouthwash and one optimal lyophilised mouthwash with good structural and mechanical properties was selected (out of five tested). The novelty in the innovative formulation of mouthwash has made it possible to use it in the prevention and treatment of, for example, periodontal disease (among the few similar products) [99].

By comparing the results obtained in our study with the results of the above-mentioned authors, we conclude that there are slight differences in AB activity, and therefore we can state that the examined plant extracts can be used for prevention and antimicrobial protection.

### 2.4. Drug Resistance Pattern of the Two Clinical Bacterial Isolates

The first clinical bacterial isolate potentially suspected to be XDR (*Klebsiella* spp.) was examined for AST against 18 commonly used antibiotics, while the *Acinetobacter baumannii complex* strain 7849 was tested against 9 antibiotics from different antimicrobial categories, according to the EUCAST standard (breakpoint tables for interpretation of MICs and zone diameters Version 14.0, valid from 1 January 2024) [92].

Most healthcare-associated infections are caused by six resistant pathogenic isolates grouped by the Infectious Diseases Society of America as ESCAPE [100]. Both isolates that we examined were classified in the ESCAPE group.

AST results of isolated *Klebsiella* spp. are shown in Table 8 and for *Acinetobacter baumannii complex* (Table 9).

*Klebsiella* spp. showed insensitivity to 17 out of 18 tested antimicrobial agents (antibiotics from different groups). Sensitivity was only proven to Cefiderocol (Ced), a novel synthetic siderophore-conjugated antibiotic that hijacks the bacterial iron transport systems facilitating drug entry into cells, achieving high periplasmic concentrations that overcome many resistance mechanisms of G− bacteria [101]. Although Cefiderocol is structurally similar to cefepime and ceftazidime [102,103], those two cephalosporin antibiotics did not show efficacy on *Klebsiella* spp. (XDR strain).

In the USA, Cefiderocol was approved in 2019 by the Food and Drug Administration (FDA) for the treatment of complicated urinary tract infections, hospital-acquired bacterial pneumonia and ventilator-associated bacterial pneumonia. In 2020, Cefiderocol was approved for the treatment of infections caused by aerobic GNB in adults with limited treatment options, after consultation with an infectious disease specialist (European Medicines Agency—EMA) [101].

*Acinetobacter baumannii* complex strain 7849, isolated from pleural punctate, showed insensitivity to 8 out of 9 tested antibiotics belonging to different classes (Table 7). In this study, this XDR microorganism showed sensitivity only to colistin (Col) from the Folate pathway inhibitor antimicrobial category. The latest views [104] regarding the effect of colistin, which has long been evaluated as the last-resort antibiotic for the treatment of infections caused by G− bacteria, force us to reconsider this opinion because more and more G− microorganisms are becoming resistant due to chromosomal mutations and the acquisition of resistance plasmid genes (mcr genes).

The escalating emergence of MDR *Klebsiella pneumoniae* strains has become a significant medical problem [105] caused by the rapid adaptation of bacteria (microorganisms) to widely used antibiotics [104]. A study by Romo-Castillo et al. [105] revealed the high potential of essential oils of thyme, rosemary and mint, as they have the ability to change the phenotype of hypermucoviscosity strains, damaging the lipid-soluble barrier that prevents the placement of antibiotics, and make its action efficient and effective, because then it could enter the bacterial cell, and, synergistically with EOs, induce bacterial death. This significant finding allows us to develop an innovative synergistic therapy against *Klebsiella pneumoniae*-induced infections, regardless of the degree of virulence, the extent of resistance or the manifestation of hypermucoviscosity.

Innovative approaches are desperately needed to overcome the serious challenge of colistin resistance. The authors anticipate research on alternative therapies, such as new antibiotics and drug combinations, where we conceptually see the importance of the research from this study [13,105,106,107,108]. Slowing the development and spread of resistance requires support for antimicrobial asset management initiatives to ensure the prudent use of colistin and other antibiotics. Thus, there is a conclusion from a number of studies that herbal compounds and bioactive molecules provide numerous benefits for the discovery, design and creation of innovative effective therapies against MDR bacterial infections. A One Health strategy can provide tools for managing colistin resistance, and planning activities related to human, animal and environmental health, with the overarching goal of effectively protecting public health for generations to come.

Based on this knowledge, and the extremely high resistance of the strains of MDR bacteria that we selected for testing, we decided to investigate the antimicrobial power of the phytocomplex mixture, justifiably expecting synergistic effects, after the primary confirmed solid antimicrobial activities on individual lyophilisates of plant raw materials (GP and DE). Thinking in the direction of the future combination of a phytocomplex cocktail, with antibiotics of proven high efficiency for potentiating the effect in already realised complex studies, represents a realistic basis for new concepts and future research within new scientific and innovative projects, especially multi- and interdisciplinary ones. A limiting factor in the use of GP was recently noticed by a group of researchers: concentrations of methanolic GP plant extract between 100 and 200 μg/mL can be considered non-toxic and antioxidative because, at higher doses (>500 μg/mL), a decrease in the viability of Caco-2 cells was demonstrated [109].

### 2.5. Total Phenolic Content and Antioxidative Capacity

It has been proven that there is a correlation between the content of total polyphenol compounds with the antioxidative activity of the DPPH radical. The content of total polyphenols in GP is 11.26 mg GAE/g of extract, while in DE, it is 22.93 mg GAE/g of plant extract. A stronger percentage of inhibition was shown by DE, with a value of 66.71%, while GP showed a percentage of inhibition at the DPPH radical level of 99.14%, which is in agreement with the concentration of total polyphenols.

The content of total polyphenols in the phytocomplex cocktail (GP + DE) is 21.22 mg GAE/g of plant extract, and the antioxidative activity expressed as a percentage of inhibition at the DPPH radical level of this sample is 69.15%, which actually indicates a linear correlation of total polyphenols with the antioxidative effect (Table 10).

DE is a plant rich in anthocyanins, with high total phenolic content and total antioxidative capacity. *Sambucus* plants are depots of abundant bioactive secondary metabolites: anthocyanins, phytosterols, flavonoids, phenols, triterpenes, tannins, iridoid glycosides, cardiac glycosides, derivatives of caffeic acid (like volatile substances), chlorogenic acid, ursolic acid and lectins [41,110,111]. The methanolic and aqueous fractions extracted from DE flowers achieved 48% and 21% inhibition in the DPPH method, respectively, so the authors conclude that DE exhibits a good DPPH radical-scavenging effect. The methanol extract from *S. ebulus* fruit is effective for its antioxidative properties on diphenyl picrylhydrazyl (DPPH) free radicals, where the antioxidative capability increases as the concentration of the extracts goes up [63,112,113].

The total phenolic content of different extracts (petroleum ether, distilled water, ethyl acetate, acetone and methanol) obtained from stems, fruits, roots and leaves of DE (collected in the region of Šumarice, Kragujevac, central Serbia) ranged from 29.87 to 126.10 mg GAE/g. The ethyl acetate extracts of fruits and plant extracts of leaves contain the highest level of non-extractable phenolic compounds and reveal an extremely strong antioxidative effect [114].

Also, the antioxidative effects of DE enable its use as protection against the teratogenic consequences of albendazole administration. Simultaneous administration of DE extract with albendazole reduces the occurrence of skeletal malformations in Wistar rats, which has been proven in animal experiments [115].

Total phenolic content in DE juice varies depending on the genotype, growth location and growing season [116], which may partially explain the differences between the values obtained for total polyphenols in our research and the results of other authors. The authors revealed the chemical composition of plant extracts prepared with various solvents: water, water with 20% co-solvent polyethylene glycol and ethanol extract as a standard. The lowest amount of selected non-extractable phenolic compounds, as well as the total polyphenols, were found in water plant extracts [117]. The extracts of fruits, flowers and leaves of *Sambucus nigra* L. and DE differ significantly in the content and composition of non-extractable phenolic compounds, which are the most important bioactive molecules with different antioxidative and antiviral effects [118]. With increasing altitude in *Sambucus nigra*, an increase in the concentration of flavonoids (especially flavonol-3-O-glycosides with adjacent hydroxyl groups in ring B) was recorded. The anthocyanin content in *Sambucus nigra* berries decreases with increasing altitude [119].

The analysed raw GP revealed a total phenolic content of 9.07 ± 0.25 mg GAE/g r.m. Dietary supplements rich in polyphenols (such as those that can be prepared from GP) may exhibit beneficial properties for human health, but it is necessary to test them for bioavailability, in in vivo studies and clinical trials, without which their toxicity and therapeutic potential cannot be evaluated [120]. Recently, alternative methods of using GP have been introduced, such as the production of extracts with antioxidative properties, and fiber extraction for the design and creation of high-value-added products enriched with bioactive molecules from GP.

Non-extractable phenolic compounds are susceptible to degradation and interaction with certain food ingredients during digestion, and encapsulation can be an extremely useful procedure for protecting the bioactivity of valuable compounds and screening their kinetics [121]. A group of Serbian authors determined a high linear correlation between different concentrations of GP extracts and their radical elimination activity (R^2^ = 0.9732), which indicates the phenolic compounds present in plant extracts that interact with the DPPH radical, and, at least partially, generate the said activity. Total phenolic content was at the level of 67.4 + 0.38 mg/g DM, total anthocyanins was 17.9 + 0.04 mg/g DM and there was a strong antioxidative effect [63].

The scavenging of DPPH radicals indicates a higher activity of GP extract from white grapes (76.33 µg/mL) than of GP extract from red grapes (112.02 µg/mL). The results of our research are in accordance with the research of Moldovan et al. [61].

Altitude significantly affects the chemical composition of grapes. Various studies reveal that an increase in altitude leads to a delay in the budding, flowering and ripening of grapes, and that cultivation at high altitudes favors a higher content of aromatic compounds and higher acidity in grapes. Also, grape skin has a higher content of anthocyanins, flavonols, total anthocyanidin monoglucosides and condensed tannins.

Other studies recorded the highest phenol content and concentration of total aromas in grapes in regions between the highest and lowest ones. Some studies indicate that growing grapes at lower altitudes leads to a higher content of condensed tannins and procyanidin compounds in grape seeds, as well as flavonols, trans-resveratrol and 3-O-acetylglucoside anthocyanins in grape skins.

It is difficult to determine how each individual variable affects grape quality, and the factors that can affect it are numerous: harvest, water or heat stress, location of the vineyard (altitude, latitude, slope and orientation), plant material (i.e., variety, clones and rootstock), training system, soil type and solar radiation. Deeper research into different grape varieties in different terroirs is necessary to enable the development of strategies for delaying grape ripening (at lower temperatures), which is important for reducing the negative effects of global warming [122].

An excellent recent overview deals with modern phytocosmetology (due to increasing demand for natural cosmetics), an extremely current field of work for research centres, which should help in the creation of such preparations that will protect the skin from free radicals and oxidative stress with minimised risk of side effects. Their review revealed the characteristics of *V. vinifera* (vine grape) species, with desirable properties for wide application in phytocosmetology, but also in the food industry and medicine [123]. A completely up-to-date review paper on the remarkable benefits of utilising GP is now available for the benefit of the scientific community [124].

## 3. Materials and Methods

### 3.1. Plant Material

GP was obtained courtesy of the owner of the Aleksandrović winery in Topola, Šumadija region, Central Serbia, at an of altitude 272 m and coordinates of 44.2547° N, 20.6782° E.

Ripe fruit of DE was picked in Krčmari village, at an altitude of about 800 m, less than 10 km from the tourist centre of Divčibare with the coordinates of 44.1556° N 19.9897° E. After harvesting, they were transported as fast as possible to the plant, for conservation (lyophilisation). The voucher specimen of DE was confirmed and deposited in the collection of the Faculty of Agriculture in Čačak, University of Kragujevac. Harvested fresh fruits are immediately used for lyophilisation. The collected plant (DE) was air-dried in darkness at room temperature.

### 3.2. Chemicals and Reagents

Folin–Ciocalteu reagent and sodium carbonate (Na_2_CO_3_) were supplied from Sigma-Aldrich^®^ (Schnelldorf, Germany); 1,1-Diphenyl-2-picrylhydrazyl free radical and Resazurin Sodium Salt (C_12_H_6_NNaO_4_) were purchased from Tokyo Chemical Industry Co., Ltd. (TCI, Tokyo, Japan); gallic acid was obtained from Acros Organic (now Thermo Fisher Scientific Inc., Fair Lawn, NJ, USA); DMSO—Dimethyl sulphoxide was obtained from Fisher Scientific International, Inc., Hampton, NH, USA; ethanol was purchased from Reahem, Novi Sad, Serbia; nutrient agar and Miller–Hinton broth (MHB) provided by the Institute of Virology, Vaccines and Sera Torlak, Belgrade, Serbia; microbiological filters FiltropurS 0.45, Lot 90245103, Sterile, were purchased from SARSTED AG & Co. KG, Nümbrecht, Germany; BD BBL Crystal Gram-positive (GP) Identification (ID) system was obtained from Becton, Dickinson, and Company (Franklin Lakes, NJ, USA), USA–BD 245240. M.Y. P agar base, Violet Red Bile Glucose Agar, Buffered Peptone Water, Salmonella Shigella Agar, Fraser Broth Base and Brilliance Listeria Agar Base were purchased from Oxoid Limited, Hampshire, UK; Tryptone Bile Glucuronic Agar, Tryptone Glucose Yeast Agar, Baird Parker Agar Base, Violet Red Bile Agar, Dichloran Rose Bengal Chloramphenicol Agar, Iron Sulpfite Agar Modified, Mueller Kauffman Tetrathionate Novobiocin Broth Base, Rappaport Vassiliadis Soya Broth, Xylose Lysine Deoxycholate Agar, Listeria Oxford Medium Base, Sodium Chloride and Tryptone Type-a were obtained from HiMedia Laboratories LLC, Kennett Square, PA, USA. Wo solvent systems were used for the gradient elution: eluent A was water with 2% HCOOH and eluent B was 80% ACN plus water with 2% acetic acid, supplied by Merck KGaA (Darmstadt, Germany). Caffeic acid, chlorogenic acid, rutin, hyperoside (que-galactoside), izoquercetin (que-glucoside), cyanidin derivative, cyanidin-3-glucoside, petunidin-3-glucoside, malvidin-acetyl-glucoside and malvidin-coumaroyl-glucoside were purchased from Sigma-Aldrich GmbH (Sternheim, Germany). Water used throughout the experiments was purified using a Millipore, Elix UV and Simplicity Water Purification System (Milford, MA, USA). All other chemicals and reagents were of analytical reagent grade.

### 3.3. Lyophilisation Procedure

Lyophilisates of plant material (DE and GP), as well as the procedure for implementing the freeze-drying process, were obtained courtesy of the owner of ELBI LLC, Valjevo, Serbia, which is engaged in the production of lyophilizer and lyophilisation of all types of food (fruit, vegetables, milk, dairy products and meat), in cooperation with the PvP centre for lyophilisation (“Lyocake” product line, https://lyocake.com/o-nama/, (accessed on 12 August 2024), Industrijska zona bb, Valjevo, Serbia. Lyophilizer LF-100 (ELBI LLC, with a capacity of 100 kg), in which the drying process was carried out under absolute vacuum, is an innovative piece of equipment because it operates on the principle of OHM Energy Saving and uses 40% less electrical energy compared to other well-known solutions, which usually represents 90% of all costs of the lyophilisation process in the ELBI LLC company’s facility.

The lyophilisation process for investigated plants was performed with the following parameters: freshly picked plants are frozen at minus 40 °C in a shock chamber not longer than 8 h after picking. After that, the frozen plants are inserted into a lyophilizer (freeze dryer), a vacuum of 0.02 mbar. This vacuum further lowers the temperature of the plant to minus 60 °C. Then, heat energy is added, which raises the temperature by 2.5 °C per hour, until the product reaches the set temperature of 38 °C, after which the product is kept for 12 to 24 h until the percentage of moisture drops to the set value (3–10%). All these parameters are set before the start of the process, and the computer creates a digital record, a diagram, which is used as proof of the correct procedure. Up to 0 °C, drying was carried out by sublimation, and above zero degrees by the classic drying process.

### 3.4. Preparation of Extracts from Lyophilisates of Plant Raw Materials (GP, DE)

Extracts were prepared for antibacterial (AB) activity testing according to the following procedure: ten grams of lyophilisates was measured for extraction, 150 mL of 50% ethanol was used, and the maceration lasted for 24 h. Supernatants collected for each of the three lyophilisates were filtered through filters (Whatman No. 1, Sigma-Aldrich, St. Louis, MO, USA), and concentrated (evaporated) in a rotary vacuum evaporator at 60 °C. After evaporating the solvent, we remove the dried plant material from the walls of the bottle with a spatula and collect the material in a dark bottle. One possible way of removing the rest of the plant extract stuck to the wall of the evaporator is to add a small amount of solvent, after which the extract is left at room temperature overnight for the solvent to evaporate. We selected and implemented a more optimal approach and evaporated the remaining solvent in a N_2_ atmosphere (Nitrogen Generator, Micro, Tremezzina, Italy). To determine AB activity, the obtained extracts were dissolved in 10% DMSO and filtered through 0.45 µm microbiological filters (FiltropurS 0.45, Lot 90245103, Sterile, SARSTED AG & Co. KG, Nümbrecht, Germany). The method used is a modification of already used methodologies for the preparation of plant extracts for microbiological analyses [123,124].

Preparation of extracts from lyophilised samples for antioxidative testing was carried out according to the following steps: extraction was performed using 80% methanol for 30 min, after which centrifugation was performed for 10 min at 5000 RPM. The weight ratio of sample to solvent was 1:10 in favor of solvent. The clear supernatants obtained by extractions were kept in the fridge, in the dark, and used for determining the total phenolic content and antioxidative activity.

Stock solutions of plant extracts for determination of AB activity were prepared by dissolving the evaporated cocktail of the phytocomplex in dimethylsulphoxide (DMSO) to the final concentration of 100 mg/mL. The serial dilutions from the stock solution were made ranging from 50 mg/mL to 0.195 mg/mL using the Mueller–Hinton broth. The described method is a modification of the applied procedure [124,125,126].

### 3.5. Chromatographic Analysis

The quantitative analysis of phenolic components was performed using the Agilent 1200 series high-pressure liquid chromatograph (HPLC) with a diode array detector (DAD) for multi-wavelength detection. The column was thermostated at 25 °C. After injecting 5 μL of the sample, the separation was performed using an Agilent Eclipse XDB C-18 (4.6 × 150 mm) column. Two solvent systems were used for the gradient elution: eluent A was water with 2% HCOOH and eluent B was 80% ACN plus water with 2% acetic acid. The applied elution program was as follows: from 0 to 10 min, 0% B; from 10 to 28 min, 25% B; from 28 to 30 min, 25% B; from 30 to 35 min, 50% B; from 35 to 40 min, 80% B; and, finally, for the last 5 min gradually decreased by 80–0% B. Compounds present in the samples were identified by the UV absorption at a wavelength range of 320–520 nm and by the retention time of analytes and reference substances. Phenolic compounds in the samples were identified by comparing their retention times and spectra with the retention time and spectra of standards for each component. Quantitative data were calculated from the calibration curves. The contents of phenolic compounds were expressed as milligrams per gram of extract (mg/g) [127].

### 3.6. Determining the Presence of Bacterial Contamination and Subsequent Isolation

The lyophilisates of the DE and GP (a by-product of wineries) were microbiologically tested for the presence of bacterial contamination using standard ISO methods as follows:

SRPS EN ISO 6579-1:2017; SRPS EN ISO 11290-1:2017; SRPS ISO 16649-2:2008; SRPS EN ISO 21528-2:2017; SRPS ISO 4832:2014; SRPS EN ISO 6888-1:2021; SRPS ISO 21527-2:2011; SRPS EN ISO 4833-1:2014+/A1:2022; SRPS EN ISO 7932:2009; SRPS CEN ISO/TS 13136:2014; and SRPS ISO 15213:2011 [75,76,77,78,79,80,81,82,83,84,85,86].

Screening for the presence of Shiga toxin-producing *E. coli* (STEC) was performed using the real-time PCR method (real-time polymerase chain reaction (PCR)-based method for the detection of the food-borne pathogens, and horizontal method for the detection of Shiga toxin-producing *Escherichia coli* (STEC) and the determination of O157, O111, O26, O103 and O145 serogroups SRPS CEN ISO/TS 13136:2014) for identifying virulence genes (*stx1*, *stx2*, and *eae*) that are associated with these strains.

Determination of the presence of *Salmonella* spp. in samples was performed in accordance with SRPS EN ISO 6579-1:2017 (horizontal method for the detection, enumeration and serotyping of *Salmonella*—Part 1: Detection of *Salmonella* spp.), which consists of four stages: pre-enrichment in a non-selective liquid medium (BPW, Oxoid, UK), selective enrichment using selective liquid Rappaport-Vassiliadis medium with soy (RVS broth Oxoid, UK) and Müller–Kauffmann Tetrathionate–Novobiocin broth (MKTTn, Oxoid, UK), selective isolation using Xylose Lysine Deoxycholate (XLD, Oxoid, UK) agar and *Salmonella Shigella* agar (SS agar, Oxoid, UK) and confirmation of suspected *Salmonella* colonies (biochemical and serological tests).

The presence of *Listeria monocytogenes* spp. was tested using a standard method SRPS EN ISO 11290-1:2017 (horizontal method for the detection and enumeration of *Listeria monocytogenes* and of *Listeria* spp.—Part 1: Detection Method). This method involves the following key steps: pre-enrichment (half-Fraser broth, Oxoid, UK), selective enrichment (Fraser broth, Oxoid, UK), plating on selective media (ALOA and Oxford agar, Oxoid, UK) and confirmation of suspected colonies (biochemical and molecular tests).

Determination of the number of enterobacteria (horizontal method for the detection and enumeration of *Enterobacteriaceae*—Part 2: Colony-count Technique—SRPS EN ISO 21528-2:2017)**,** coliform bacteria (horizontal method for the enumeration of coliforms—colony-count technique—SRPS ISO 4832:2014), *E. coli* (horizontal method for the enumeration of beta-glucuronidase-positive *Escherichia coli*—Part 2: Colony-count Technique at 44 Degrees C using 5-bromo-4-chloro-3-indolyl beta-D-glucuronide—SRPS ISO 16649-2:2008) and aerobic colony count (horizontal method for the enumeration of microorganisms—Part 1: Colony Count at 30 °C and Amendment 1: Clarification of Scope—SRPS EN ISO 4833-1:2014+/A1:2022) was performed using the pour plate technique.

Quantifying sulphite-reducing bacteria was also performed using the pour plate method with anaerobic incubation (horizontal method for the enumeration of sulphite-reducing bacteria growing under anaerobic conditions—SRPS ISO 15213:2011).

Determining the number of coagulase-positive staphylococci (horizontal method for the enumeration of coagulase-positive staphylococci (*Staphylococcus aureus* and other species)—Part 1: Method using Baird-Parker Agar Medium—SRPS EN ISO 6888-1:2021), *Bacillus cereus* (horizontal method for the enumeration of presumptive *Bacillus cereus*—colony-count technique at 30 °C—SRPS EN ISO 7932:2009), yeasts and moulds (horizontal method for the enumeration of yeasts and moulds—Part 2: Colony-count Technique in Products with Water Activity Less Than or Equal to 0.95—SRPS ISO 21527-2:2011) was performed using the surface plating technique.

If the presence of bacteria was determined, the next step was identification. Samples were prepared according to the manufacturer’s instructions, in this case, BD BBL Crystal Gram-positive (GP) Identification (ID) system (Becton, Dickinson, and Company, USA—BD 245240), designed to identify aerobic Gram-positive bacteria.

All of the above methods are in accordance with the Law on Food Safety (Official Gazette No. 41/2009) and related by-laws and reports on food safety, harmonised with European regulations (Regulation of the EC Commission 2073/2005 criteria for foodstuffs).

All microbiological analyses were performed at the Department for Testing Raw Materials of Animal Origin, Food and Water (Sector for Laboratory Testing) of the Veterinary Specialist Institute “Kraljevo”, Kraljevo, Serbia (VSIKV).

### 3.7. AB Activity

Standard eight bacterial strains (*Listeria monocytogenes* ATCC 13932, *Bacillus subtilis* ATCC 6633, *Pseudomonas aeruginosa* ATCC 27853, *Proteus mirabilis* ATCC 35659, *Salmonella enteritidis* ATCC 13076, *Enterococcus faecalis* WDCM 00009 product number VT 000096 Vitroids™, *Staphylococcus aureus* ATCC 25923 and *Escherichia coli* WDCM 00012 Vitroids™) were used for the microdilution method (determination of MIC/MBC).

The minimum inhibitory concentration (MIC) and the minimum bactericidal concentration (MBC) were determined by the microdilution method with resazurin as an indicator. The procedure includes preparation of McFarland standard, inoculum, resazurin solution and determination of MIC/MBC. An amount of 50 µL of Miller Hinton nutrient broth (MHB) was introduced and dispensed into each of the 96 wells of the microtiter plate, and 50 µL of the solution of the tested extract was added to the first row of the plate. Then, the double dilution method was applied. After that, 50 µL of bacterial suspension was added (the final concentration of bacteria in the well is 5 × 10^6^ CFU/mL). Resazurin (TCI, Tokyo, Japan) was used as an indicator to determine the MIC, 10 µL of resazurin solution was added to each well inoculated with bacteria [128]. The test was performed in three replicates, with a mandatory negative control (MHB only, sterility control) and a positive control (MHB + bacterial inoculum). Minimal bactericidal concentration (MBC) was determined as follows: 10 µL of the well content was inoculated onto the MHB plate. After 24 h of incubation, the MBC value is considered the lowest concentration in which no growth was observed.

### 3.8. Examination of Clinical Samples from Patients with Suspected Healthcare-Associated Infections: Isolation, Identification and Characterisation of Bacterial Isolates

The two samples from clinical patients (healthcare-associated infections, from urine and pleural punctate) from the General Hospital in Čačak (Morava district) were tested in the Microbiological Laboratory of the Department of Public Health in Čačak, Serbia, and XDR bacterial strains were isolated, identified and characterised using standard biochemical tests and the commercial identification systems. The following microorganisms were confirmed using VITEK^®^ 2 gram-negative (GN) identification cards (bioMerieuk, Inc., Durham, NC, USA):

1. *Klebsiella* spp. (from urine);

2. *Acinetobacter* baumannii complex (from pleural punctate).

### 3.9. Antimicrobial Susceptibility Testing (AST) of Characterised Clinical Bacterial Isolates by Performing the DD and Microdilution Method (Determination of MIC)

AST of the isolated *Klebsiella* spp. and *Acinetobacter baumannii complex* was performed using the DD test. AST was performed using standard AST antibiotic-impregnated discs (Bio-Rad Laboratories, Hercules, CA, USA). Bacterial culture suspensions were prepared in saline at a density of 0.5 McFarland. Sterile swab suspensions were inoculated onto Mueller–Hinton agar plates (Oxoid Limited, Basingstoke, UK). After inoculation, discs were placed on the 90 mm diameter MH agar plate (maximum 5 per plate). Inoculated disk plates were incubated at 35 °C ± 2 °C for 18 h. After the predicted optimal time, the zones of inhibition were measured, with which the sensitivity of the bacteria was determined. Categorisation and interpretation of results are performed in accordance with the standard in use (S, sensitive; I, medium; R, resistant) [128].

The AST of both clinical isolates (*Klebsiella* spp. and *Acinetobacter baumannii complex*) must be determined exclusively by the microdilution method, i.e., by quantitative determination of the MIC of colistin against the non-fastidious Gram-negative bacteria (G−) (*Enterobacterales*, *Pseudomonas aeruginosa* and *Acinetobacter* spp.). ComASP Colistin 0.25–16 system for colistin susceptibility testing with broth microdilution method was used, as recommended by the manufacturer’s instructions (Liofilchem, Roseto degli Abruzzi, Italy, Rev. 4/29.09.2023). After preparing the suspension and standardising the tested bacteria to the density of a McFarland 0.5 standard, 15 min after preparation, the adjusted suspension was diluted 1:20 in saline (Solution A). Then, 0.4 mL of Solution A was added to the MH II Broth tube, provided in the kit to obtain Solution B. Next, 100 µL of Solution B was dispensed into each well in a row. The panel was covered with a lid and incubated at 36 + 2 for 16–20 h in ambient air. The MIC represents the lowest concentration of colistin that inhibited the growth of both test bacteria, visible as turbidity or as a button at the bottom of the well. MIC values were interpreted in accordance with current EUCAST criteria [128].

### 3.10. Determining the Total Phenolic Content and Antioxidative Activity at the Level of DPPH Radicals

#### 3.10.1. Determining the Total Phenolic Content

Total phenolic content was determined by a modified Folin–Ciocalteu colorimetric method [129]. An amount of 40 µL of extract or gallic acid standard solution was mixed with 3.16 mL of distilled water, and then 200 µL of Folin–Ciocalteu reagent was added. After 8 min, 600 µL of 20% Na_2_CO_3_ solution was added, and the solution was mixed well and left to incubate for 2 h at room temperature. After incubation, the absorbance was measured at a wavelength of 760 nm using a UV-VIS spectrophotometer (Cary Series 300 Agilent Technologies, Santa Clara, CA, United States). GAE was used to create the standard curve; the results are expressed in milligrams of gallic acid equivalents per g (mg GAE/g) of extract and tabulated (Table 9). Analyses were performed by software Statistica 12.5 (StatSoft, Inc., Tulsa, OK, USA) and presented as a mean ± standard deviation (SD). Differences between means were determined using Tukey’s HSD test at the significance level of *p* < 0.05.

#### 3.10.2. DPPH Method

The antioxidative capacity was determined by evaluating the free radical-scavenging effects on the 1,1-diphenyl-2-picrylhydrazyl (DPPH) radical. An aliquot of 0.1 mL of the extract was mixed with 3.9 mL of DPPH solution (6 × 10^−5^ mol∙dm^−3^) and the mixture was thoroughly vortexed and left in a dark room for 30 min. This was followed by the measurement of absorbance at 515 nm, with distilled water used as a “blank”. The results are expressed as a percentage of DPPH radical inhibition, and were calculated according to the following equation:

DPPH scavenging effect % = [(AB − AA)/AB] × 100

where

AB is the absorbance of DPPH solution without extracts;
AA is the absorbance of DPPH solution with extracts.

### 3.11. Determining the Activity of Water (a_w_) of Plant Raw Material Lyophilisates

The a_w_ of the samples was determined according to the standard method ISO 18787:2017 (determination of water activity in foodstuffs) [130] using the Lab Master Basic device, Novasina AG, Switzerland. The sample was placed in appropriate containers filled up to 2/3 of the height, and the containers were then placed in the measuring chamber of the apparatus. The measurement process was performed at a constant temperature of 20 °C. Results of a_w_ values are presented as a mean ± standard deviation (SD) and differences between means were determined using Tukey’s HSD test (at the significance level *p* < 0.05) performed by software Statistica 12.5 (StatSoft, Inc., Tulsa, OK, USA).

## 4. Conclusions

A cocktail of phytocomplexes can be used as a conventional medicine or food supplement, with recommendations for the possibility of their use as an antimicrobial agent for the treatment of infections with MDR bacteria, and even XDR bacterial strains. They represent potential natural nutraceuticals. This study enables the continuation of the successful design and creation of different reformulations of healthier or functional foods with improved functional and nutritional properties of natural (herbal) nutritional supplements. On the other hand, this study opens up possibilities for interdisciplinary research and the creation of pharmaceutical or cosmetic preparations. To date, there is no complete agreement on the superior reference models containing the definition of combined effects, although there are models used in the research of herbal medicines and potential natural food preservatives. This study is very interesting because there is no data from the literature in scientific circles with a clearly defined chemical composition or antimicrobial and antioxidative activity of the phytocomplex mixture prepared from GE/DE. The natural products community can and must initiate the development of effective strategies that would implement an effective safety and toxicity assessment of phytocomplex mixtures, which would in turn encourage multidisciplinary project-based professional and scientific research.

## Figures and Tables

**Table 1:** Analytical parameters for 9 phenolic compounds used for HPLC-DAD analysis.

Compound	Calibration Curve	(*R*^2^)	LOD µg/mL	LOQ µg/mL
Caffeic acid	y=33525.3x+0.43	0.9998	0.08	0.26
Chlorogenic acid	y=10525.5x+0.12	0.9992	0.01	0.04
*p*-Coumaric acid	y=16622.1x+0.33	0.9997	0.04	0.14
Rutin	y=4315.3x+0.23	0.9995	0.05	0.17
Hyperoside	y=5325.7x+0.08	0.9990	0.03	0.10
Isoquercetin	y=4617.8x+0.12	0.9996	0.03	0.10
Cyanidin-glucoside	y=5.725.1x+0.09	0.9992	0.03	0.10
Petunidin-glucoside	y=5721.0x+0.11	0.9992	0.02	0.07
Malvidin-glucoside	y=5023.2x+0.22	0.9995	0.02	0.07

R—Regression Coefficient; LOD—Limit of Detection; LOQ—Limit of Quantification.

**Table 2:** Phenolic profile of Grape Pomace (GP) extract.

Compound	Content (mg/g)
Malvidin-3-glucoside	2.388
Malvidin-acetyl-glucoside	0.359
Petunidin-3-glucoside	0.268
Malvidin-coumaroyl-glucoside	0.174
Summary	3.189

**Table 3:** Phenolic profile of Dwarf Elder (DE) extract.

Compound	Content (mg/g)
Cyanidin derivative	3.730
Hyperoside (que-galactoside)	2.319
Rutin	0.944
Isoquercetin (que-glucoside)	0.909
Caffeic acid	0.468
Chlorogenic acid	0.235
Summary	8.605

**Table 4:** Polyphenol compounds of phytocomplex (GP + DE).

Phytocomplex GP + DE	Content (mg/g)
Cyanidin-3 glucoside	4.134
Hyperoside (que-galactoside)	2.454
Petunidin-3-glucoside	2.348
Rutin	0.996
Isoquercetin (que-glucoside)	0.991
Caffeic acid	0.497
Chlorogenic acid	0.241
Malvidin-coumaroyl-glucoside	0.192
*p*-Coumaric acid	0.144
Cyanidin derivative	0.059
Cyanidin derivative	0.049
Malvidin-acetyl-glucoside	0.045

GP—Grape Pomace, DE—Dwarf Elder.

**Table 5:** Presence of bacterial contamination in samples of various plant lyophilisates.

Samples (Lyophilised Plants)	Parameters	Unit of Measure	Results	Method	Found Contaminants
Grape Pomace (GP)	Aerobic colony count	cfu/g	170	SRPS EN ISO 4833-1:2014 [75]	None
	Enterobacteriaceae	cfu/g	<10	SRPS EN ISO 21528-2:2017 [76]	
	Coagulase-positive staphylococci (CNS)	cfu/g	<10	SRPS EN ISO 6888-1:2021 [77]	
	*Salmonella* spp.	in 25 g	Not detected	SRPS EN ISO 6579-1:2017 [78]	
	Total coliforms	cfu/g	<10	SRPS ISO 4832:2014 [79]	
	*Listeria monocytogenes*	in 25 g	Not detected	SRPS EN ISO 11290-1:2017 [80]	
	*Escherichia coli*	cfu/g	<10	SRPS ISO 16649-2:2008 [81]	
	Yeasts	cfu/g	<10	SRPS ISO 21527-2:2011 [82]	
	Moulds	cfu/g	<10	SRPS ISO 21527-2:2011 [83]	
	*Bacillus cereus*	cfu/g	<10	SRPS EN ISO 7932:2009 [84]	
	Sulphite-reducing clostridia	cfu/g	<10	SRPS ISO 15213:2011 [85]	
	Shiga toxin-producing *Escherichia coli* (STEC)	in 25 g	Not detected	SRPS CEN ISO/TS 13136:2014 [86]	
Danewort, Dwarf Elder (*Sambucus ebulus* L.) (DE)	Aerobic colony count	cfu/g	1700	SRPS EN ISO 4833-1:2014 [75]	None
	Enterobacteriaceae	cfu/g	<10	SRPS EN ISO 21528-2:2017 [76]	
	Coagulase-positive staphylococci (CNS)	cfu/g	<10	SRPS EN ISO 6888-1:2021 [77]	
	*Salmonella* spp.	in 25 g	Not detected	SRPS EN ISO 6579-1:2017 [78]	
	Total coliforms	cfu/g	<10	SRPS ISO 4832:2014 [79]	
	*Listeria monocytogenes*	in 25 g	Not detected	SRPS EN ISO 11290-1:2017 [80]	
	*Escherichia coli*	cfu/g	<10	SRPS ISO 16649-2:2008 [81]	
	Yeasts	cfu/g	60	SRPS ISO 21527-2:2011 [82]	
	Moulds	cfu/g	<10	SRPS ISO 21527-2:2011 [83]	
	*Bacillus cereus*	cfu/g	<10	SRPS EN ISO 7932:2009 [84]	
	Sulphite-reducing clostridia	cfu/g	<10	SRPS ISO 15213:2011 [85]	
	Shiga toxin-producing *Escherichia coli* (STEC)	in 25 g	Not detected	SRPS CEN ISO/TS 13136:2014 [86]	

**Table 6:** a_w_ values of samples of various tested plants lyophilisates.

Tested Samples (Lyophilised Plants)	GP	DE
a_w_ values	0.310 ± 0.002 ^a^	0.257 ± 0.005 ^b^

^a,b^ Values (mean ± SD) with different superscripts are significantly different (*p* < 0.05). GP—Grape Pomace, DE—Dwarf Elder.

**Table 7:** MIC and MBC of plant extracts (mg/mL).

Tested Bacterial Strains (Conventional, Standard Strains)	GP		DE		COCKTAIL (GP + DE)	
	MIC	MBC	MIC	MBC	MIC	MBC
*Listeria monocytogenes* ATCC 13932	3.125	>6.250	6.250	>12.500	3.125	>12.500
*Bacillus subtilis* ATCC 6633	6.250	>12.500	6.250	>12.500	3.125	>12.500
*Pseudomonas aeruginosa* ATCC 27853	12.500	>25.000	12.500	>25.000	6.250	25.000
*Proteus mirabilis* ATCC 35659	12.500	>25.000	12.500	>25.000	6.250	25.000
*Salmonella enteritidis* ATCC 13076	6.250	>12.500	6.250	>12.500	6.250	>12.500
*Enterococcus faecalis* WDCM 00009 VT 000096 *vitroides*	0.780	>1.560	3.125	>6.250	3.125	>12.500
*Staphylococcus aureus* ATCC 25923	3.125	>6.250	3.125	>6.250	3.125	>12.500
*Escherichia coli* WDCM 00012 *vitroides*	6.250	>12.500	6.250	>12.500	3.125	>12.500
XDR bacteria						
*Klebsiella* spp.	/	/	/	/	12.500	>50.000
*Acinetobacter baumannii* complex	/	/	/	/	6.250	50.500

**Table 8:** Susceptibility of *Klebsiella* spp. on certain antimicrobial agent (DD method). ** Col-MIC.

Strain Designation	AMA																	
	Gen	Ami	Tob	Pip	Ert	Ced	Imi	Mer	Chl	Cer	Cef	Cep	Cip	Lev	Tri	Amp	Acl	Col
7840	R	R	R	R	R	S	R	R	R	R	R	R	R	R	R	R	R	R

* Antimicrobial category; ** Antimicrobial agents; * Aminoglycosides. ** Gen, Gentamicin; Ami, Amikacin; Tob, Tobramicin; * Anti-pseudomonas penicillin’s + β-lactamases inhibitors. ** Pip, Piperacillin Tazobactam; * Carbapanems. ** Ert, Ertapenem; Imi, Imipenem; Mer, Meropenem; * Cephalosporin’s I and II generation. ** Chl, Cephalexin; * Cephalosporin’s III and IV generation. ** Cer, Ceftriaxone; Cef, Ceftazidime; Cep, Cefepime; ** Ced, Cefiderocol; * Fluorochinolones. ** Cip, Ciprofloxacin; Lev, Levofloxacin; * Folate pathway inhibitors. ** Tri, Trimethoprim/sulfamethoxazole; * Penicillins. ** Amp, Ampicillin. * Penicillin’s + β-lactamases inhibitors. Acl, Amoxicillin-clavulanate; * Polymixins. ** Col, colistin; S: susceptible; R: resistant.

**Table 9:** Susceptibility of *Acinetobacter baumannii complex* on certain antimicrobial agent (DD method). ** Col-MIC.

Strain Designation	AMA								
	Gen	Ami	Tob	Imi	Mer	Cip	Lev	Tri	Col
7849	R	R	R	R	R	R	R	R	S

* Antimicrobial category; ** Antimicrobial agents; * Aminoglycosides. ** Gen, Gentamicin; Ami, Amikacin; Tob, Tobramicin; * Anti-pseudomonas carbapanems. ** Imi, Imipenem; Mer, Meropenem; * Ant pseudomonas Fluoroquinolones. ** Cip, Ciprofloxacin; Lev, Levofloxacin; * Folate pathway inhibitors. ** Tri, Trimethoprim Sulfamethoxazole; * Polymixines. ** Col, colistin; S: susceptible; R: resistant.

**Table 10:** Total phenolic content and the scavenging of DPPH radicals’ activity of various tested lyophilisates and phytocomplex (a cocktail of Grape Pomace and Dwarf Elder).

Plant Material (Lyophilisates)	Total Phenolic Content (mg GAE/g)	DPPH (Inhibition, %)
Grape Pomace	11.26 ± 0.98 ^b^	92.14 ± 1.13 ^a^
Danewort, Dwarf Elder (*Sambucus ebulus* L.)	22.93 ± 0.21 ^a^	66.71 ± 1.17 ^b^
Phytocomplex (cocktail of Grape Pomace and Dwarf Elder)	21.22 ± 1.20 ^a^	69.15 ± 1.39 ^b^

All data are expressed as means value of replication (n = 3) ± SD. ^a,b^ Values (mean ± SD) in the same column with different superscripts are significantly different (*p* < 0.05).

## Data Availability

The original contributions presented in the study are included in the article, and further inquiries can be directed to the corresponding author.
